# Directional light beams by design from electrically driven elliptical slit antennas

**DOI:** 10.3762/bjnano.9.221

**Published:** 2018-09-03

**Authors:** Shuiyan Cao, Eric Le Moal, Quanbo Jiang, Aurélien Drezet, Serge Huant, Jean-Paul Hugonin, Gérald Dujardin, Elizabeth Boer-Duchemin

**Affiliations:** 1 Institut des Sciences Moléculaires d’Orsay (ISMO), CNRS, Univ Paris Sud, Université Paris-Saclay, F-91405 Orsay, France; 2 Université Grenoble Alpes, Institut NEEL, F-38000 Grenoble, France and CNRS, Institut NEEL, F-38042 Grenoble, France; 3 Laboratoire Charles Fabry, Institut d’Optique, 91127 Palaiseau, France

**Keywords:** elliptical antenna, inelastic electron tunneling, optical antenna, plasmonics, scanning tunneling microscopy, surface plasmon polariton

## Abstract

We report on the low-energy, electrical generation of light beams in specific directions from planar elliptical microstructures. The emission direction of the beam is determined by the microstructure eccentricity. A very simple, broadband, optical antenna design is used, which consists of a single elliptical slit etched into a gold film. The light beam source is driven by an electrical nanosource of surface plasmon polaritons (SPP) that is located at one focus of the ellipse. In this study, SPPs are generated through inelastic electron tunneling between a gold surface and the tip of a scanning tunneling microscope.

## Introduction

With the ever-growing demand for higher information capacity and the diversification of applications, the integration of nanophotonics with nanoelectronics in microdevices has never been more relevant than now [1–9]. In this context, miniaturized electrical light sources are needed for chip-to-chip communication and optical interactions with the surrounding environment (e.g., for remote control, external communication or sensing applications). A number of electrically driven emitting optical antennas have been described in the recent literature [10–19], where the emission of light is activated using low voltage (a few volts) and low current (nanoamperes to microamperes), compatible with integrated electronics. These antennas were based on plasmonic micro- or nanostructures of various geometries (Yagi–Uda, bull’s-eye, nanoparticle dimer, or wire antennas), coupled in the near field (or incorporating in their design) an electrically driven nanosource of surface plasmon polaritons (SPPs, light waves coupled to electron density oscillations at a metal–dielectric interface). In particular, the electrical SPP nanosource can be a nanoscale tunnel junction where the emission process relies on inelastic electron tunneling effects [20].

A central issue for miniaturized electrical light sources is the control of their emission direction, especially given that SPP excitation with electrons results in a broad power spectrum [21–24]. When based on a single plasmonic nanoparticle, these sources exhibit angularly broad emission patterns resembling that of an electric dipole, possibly with additional higher-order multipolar contributions [25–27]. Antenna designs based on the arrangement of several plasmonic nanoparticles (e.g., Yagi–Uda antennas [28]) yield more directional emission [29]. However, the light is most often emitted at the critical angle of the substrate–superstrate interface [30] or in directions that strongly vary depending on the optical frequency [31]. Light beams with an angular spread of only a few degrees [32–33] may be obtained from periodic microstructures (e.g., bull’s-eye antennas [34–35]), yet again with a strong dependence on frequency. Foremost, planar antennas with cylindrical symmetry, such as bull’s-eye [36] and patch antennas [37–38], invariably emit light in a beam or cone the axis of which is orthogonal to the surface plane. Thus, for all applications where light is emitted from electrically driven optical antennas and specific directions of emission are desired, a different antenna design has to be found [39].

Recently, we have demonstrated that the electrical excitation of SPPs in the center of a plasmonic lens consisting of a single circular slit etched in a gold film results in the emission of a spectrally broad cylindrical vector beam of light [40–41]. The resulting emission direction, which is invariably orthogonal to the surface plane on average, does not depend on the frequency and the angular spread of the resulting beam is determined by the ratio of the lens diameter to the emission wavelength. In addition, previous experiments carried out using a scanning electron microscope in vacuum have shown that oblique light beams may be produced from elliptical corrals etched in a single crystal of gold. In order to produce the oblique beam of light, the high-energy (30 keV) electron beam is focussed onto one of the two focal points of the structure [42]. The emission direction is determined by the eccentricity of the corral and could, in principle, be tailored by design. Inspired by this work, we investigate in the present paper the low-energy (below 3 eV) electrical excitation and the resulting light beams from single elliptical antennas consisting of an elliptical slit etched in a gold film. We theoretically and experimentally show that when the excitation takes place at one of the two focii, these elliptical slits act as highly directional antennas that convert electrically excited SPPs into light beams that are emitted in specific directions as determined by the ellipse eccentricity and the refractive index of the surrounding medium. The angular spread of the emitted beam is inversely proportional to the length of the ellipse axes.

## Results and Discussion

**Figure 1:**
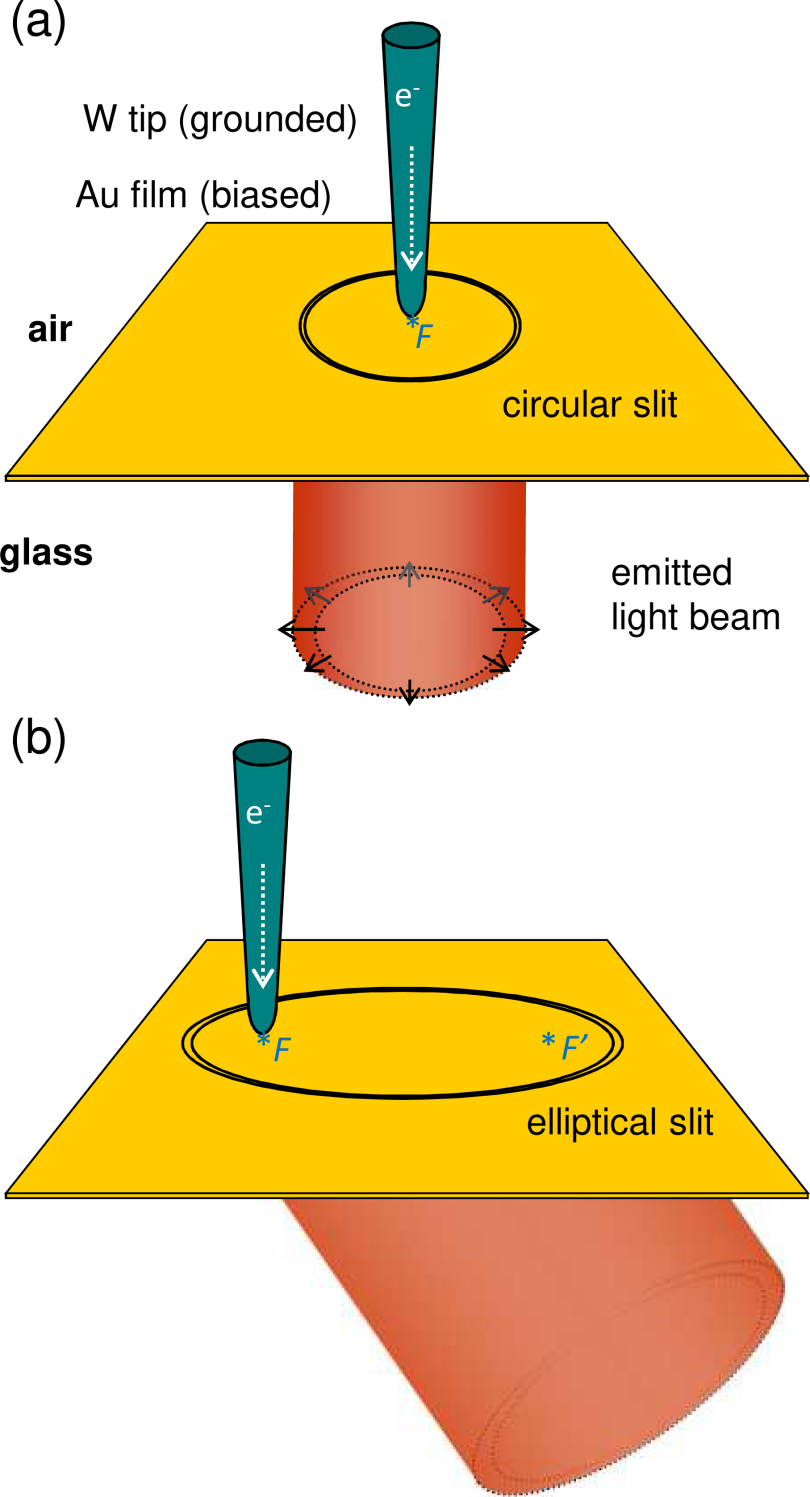
Schematics of the experiment. A single (a) circular or (b) elliptical slit etched in a 200 nm gold film on glass is used as an electrical light beam microsource. Inelastic electron tunneling from the tungsten tip of an STM excites SPPs at the air-gold interface. These SPPs propagate isotropically away from the tip to the slit where they scatter into light. Excitation at (a) the center of the circle or (b) at a focus of the ellipse yields (a) radially polarized cylindrical vector beams orthogonal to the plane or (b) oblique light beams in a specific direction as determined by the eccentricity of the ellipse.

Figure 1 shows schematics of the experiment performed in this study. All experiments are carried out in air and at room temperature using a scanning tunneling microscope (STM) head mounted on top of an inverted optical microscope. The setup is described in detail in the Experimental section. Circular or elliptical slits are etched in an optically thick (200 nm) gold film deposited on a glass coverslip. The inelastic effects of the tunnel current between the STM tip and the surface of the gold film generate circular waves of surface plasmon polaritons, which propagate isotropically away from the tip along the air–gold interface [23]. The SPPs are scattered at the slit into photons in air (not shown) and in the glass. Only the light emitted in the glass is collected using a high numerical aperture (NA) microscope objective. The angular distribution of the emitted light is acquired from Fourier-space images [24–25].

The angular emission pattern results from the far-field interference of the light scattered from all along the slit. When the STM tip is positioned in the center of the disc formed by a circular slit (see Figure 1a), all points along the perimeter of the slit are equidistant from the SPP source. Thus, light is emitted in phase from all along the slit. As a result, a light beam is emitted in the direction orthogonal to the surface plane. Due to the cylindrical symmetry of the system, the emitted beam is a cylindrical vector beam with radial polarization [40].

When the tip is off-center, the emission from different positions along the circular slit is out-of-phase. As previously reported in [40], light beams tilted by up to 10° may be obtained in this way while maintaining a comparatively low angular spread. However, beyond this limit more intricate emission patterns are obtained. A lateral shift of the excitation source is not equivalent to an angular tilt of the emitted beam. Different geometries must be found in order to produce light beams in specific directions.

### The working principle of an elliptical slit antenna

**Figure 2:**
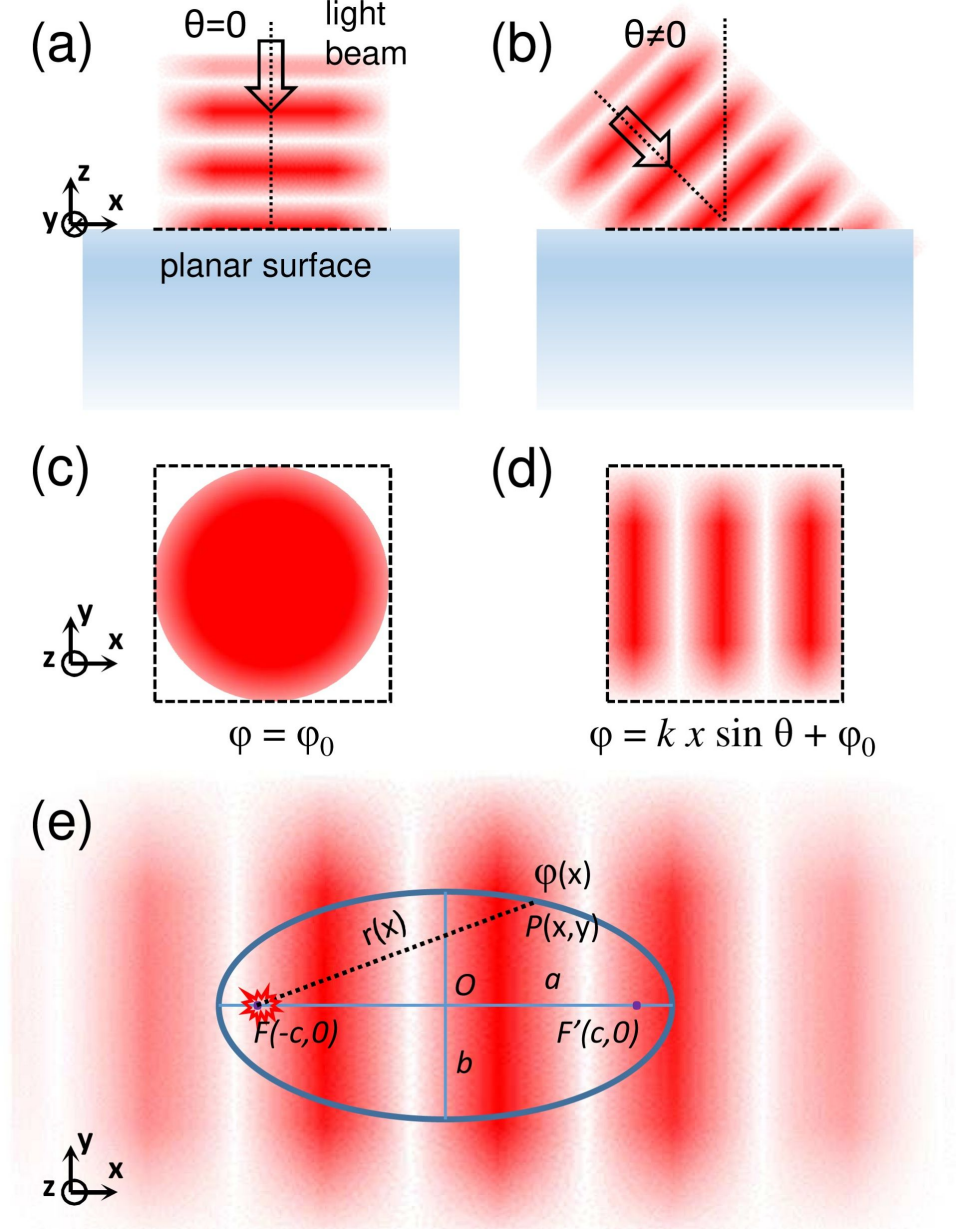
Working principle of an elliptical slit antenna: control of the phase. At a planar surface, the phase of the electric field of an incident light beam in (a,c) orthogonal or (b,d) oblique incidence is spatially (a,c) independent or (b,d) dependent, respectively. If this spatial phase distribution is reproduced on a planar surface, an extended planar light source can emit a beam of light in a particular direction out of the plane. (e) Such a light source is obtained when an elliptical slit antenna is fed by circular surface waves at one of its focii. The scattered light from the slit has the same spatial phase distribution at the surface as a light beam arriving at oblique incidence. The eccentricity of the ellipse determines the emission angle of the beam.

Figure 2 shows a planar surface illuminated by a plane wave light beam. If the incidence angle is θ = 0, the optical field at the surface is in phase for the entire illuminated area. Otherwise, if θ≠ 0 and the incidence plane is the **xz**-plane, the phase φ of the optical field at the surface varies with position, i.e., φ(*x*) = *kx*sin θ + φ_0_, where *k* is the wavevector modulus of the incident light and φ_0_ is a constant. In order to emit a beam of light in a specific direction, a light source at a planar surface must reproduce this spatial phase distribution. In the specific case of a slit that scatters surface waves (emitted from a point-like source) into light, the phase is the delay due to the propagation of the surface waves from the source to the slit. Thus, the distance *d* traveled must vary along the slit such that the phase of the scattered light is

[1]
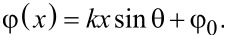


We assume that the propagation and scattering of these surface waves may be treated within the scalar approximation as in the case of plane waves in free space. This approximation has been shown to be correct for the case of SPPs [43]. Within this approximation, Equation 1 reads κ*d* = *kx*sin θ + φ_0_, where κ is the wavevector modulus of the surface waves.

The only geometry that meets this condition is an ellipse with the surface wave source located at one of the focii. An ellipse is the ensemble of points *P*(*x*,*y*) that satisfies the equation (*x*^2^)/(*a*^2^) + (*y*^2^)/(*b*^2^) = 1, where *a* and *b* are the semiaxes of the ellipse. If *a > b* and the ellipse is centered at (0,0), the focii are at *F*(−*c*,0) and *F*′(*c*,0), where *c*^2^ = *a*^2^ − *b*^2^. The eccentricity of the ellipse is *e* = *c*/*a*. The distance from *F* to *P*(*x*,*y*) is *r* = *ex* + *a*. As a result, when the source is at *F*, wave propagation to the slit introduces a phase shift of κ(*ex* + *a*). Thus, a light beam emitted at an angle θ is expected where θ satisfies the equation

[2]
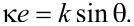


In this way the emission angle may be tailored by varying the eccentricity of the ellipse. The available angular range is from θ = 0 to θ = arcsin (κ*e*)/*k*, with *e* chosen between 0 (i.e., a circle) and up to but not including 1 (*e* = 1 yields a parabola).

**Figure 3:**
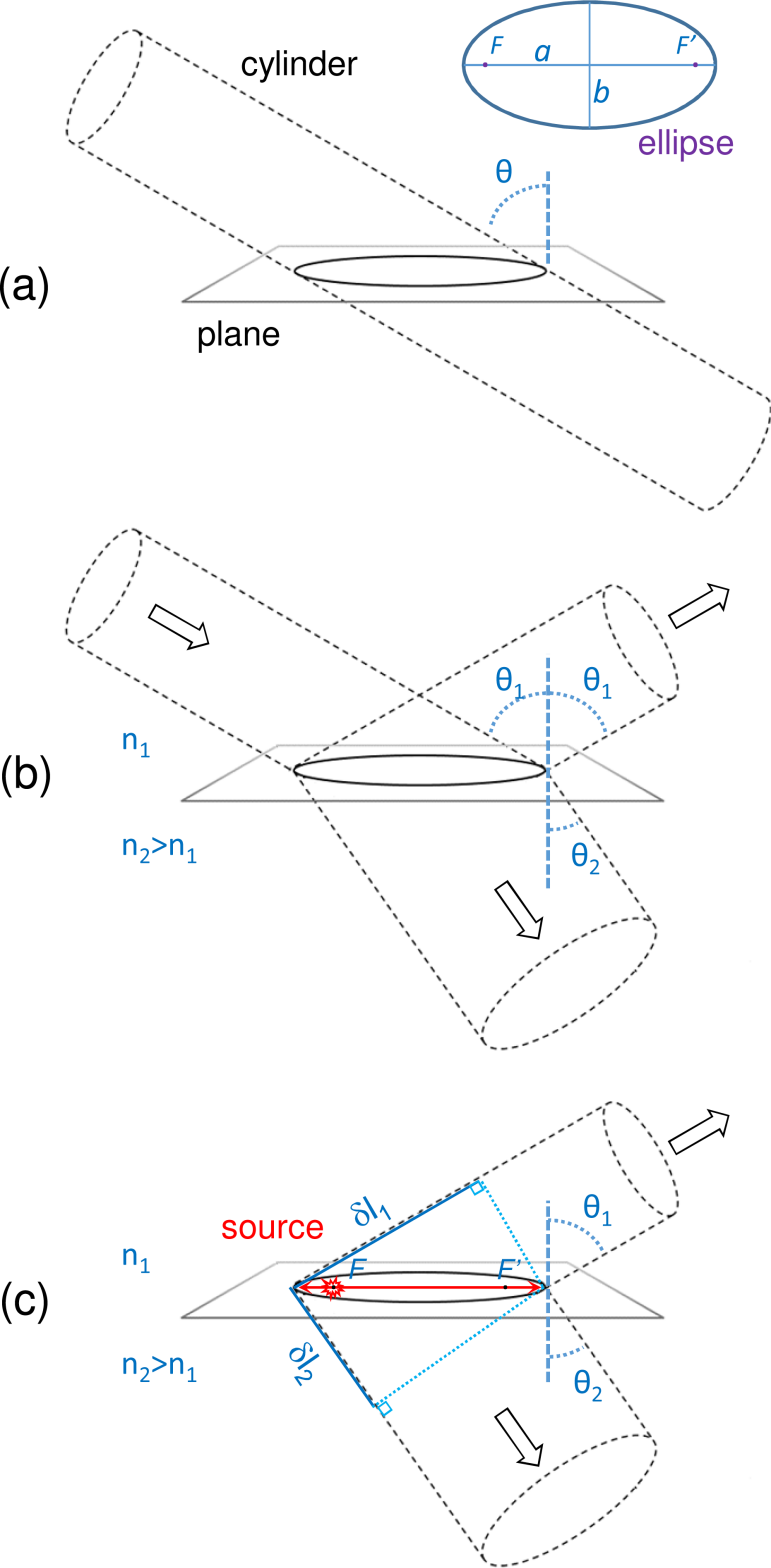
Working principle of an elliptical slit antenna: shape of the emitted beams. (a) The intersection of a cylinder and a plane is an ellipse. However, elliptical slit antennas do not necessarily emit cylindrical beams. (b) This is in analogy to a cylindrical beam with a circular section that becomes elliptical after refraction at the interface between two media with different refractive indices. (c) Similarly, due to different propagation speeds of light in the superstrate, of light in the substrate and of the surface waves at the interface, elliptical slit antennas emit non-cylindrical light beams at angles that depend on the refractive indices of the upper and lower media.

Figure 3a shows that the intersection of a cylinder with a plane yields an ellipse the eccentricity of which is precisely *e* = sin θ, where θ defines the tilt of the cylinder axis with respect to the normal of the plane. Based on the similarity with Equation 2, one may expect that cylindrical beams can be emitted from elliptical slit antennas fed with surface wave sources at their focii. However, Equation 2 shows that this is only true if light and surface waves propagate at the same speed, i.e., κ = *k*. Figure 3b shows that the refraction of a cylindrical beam at a planar interface between two media does not yield a cylindrical beam if the two media do not have same index of refractive, since the shape of the beam changes as a result of the change in emission angle. In the case of reflection, since the medium and thus the emission angle remain the same, the beam remains cylindrical.

In order to have a beam of light, the phase due to the optical path difference of light in the medium (i.e., *k*_0_*n*_1_δ*l*_1_ or *k*_0_*n*_2_δ*l*_2_) must equal the phase due to the difference in propagation length from the source at a focus to the slit (i.e., 2κ*c*). *k*_0_ is the wavevector modulus of photons in vacuum. In other words, 2(κ/*k*_0_)*c* = *n*_1_δ*l*_1_ = *n*_2_δ*l*_2_. Air and glass have refractive indexes *n*_1_ and *n*_2_ of about 1 and 1.5, respectively, and the SPPs at the air–gold interface have an effective index close to 1. Our elliptical slit antennas are thus expected to emit quasi cylindrical beams on the air side, since the speed of photons in air, and of SPPs on an air–gold interface are similar. In contrast, the light beam emitted in glass must have an elliptical section. This is not directly visible in the Fourier-space images since they reveal the angular, and not the spatial distribution of the emitted light. Note that the model of a cylinder intersecting a plane introduced above yields similar results as the virtual parabola model proposed in [42] for the beaming of an elliptical cavity and provides a simple framework to describe the shape of the emitted beams.

We can produce beams of light emitting in the angular range from 0° to arcsin(*k*_SPP_/*n_i_k*_0_), with *i* = 1 in air and *i* = 2 in glass. In principle, our elliptical slit antennas can thus emit light beams at all polar angles in air (i.e., 0° to about 90°) and from 0° to about 43° in glass (at λ_0_ = 700 nm). Interestingly, light beams at virtually all polar angles in glass (i.e., up to 90°) could be obtained if the SPP nanosource were located at the glass–gold interface or if it excited a gold film sandwiched between two media of the same refractive index (e.g., a suspended gold membrane in air). The latter geometry (i.e., a prototypical insulator–metal–insulator waveguide) has the additional advantage that it supports long-range SPPs that have lower propagation losses [44].

### Angular emission pattern

**Figure 4:**
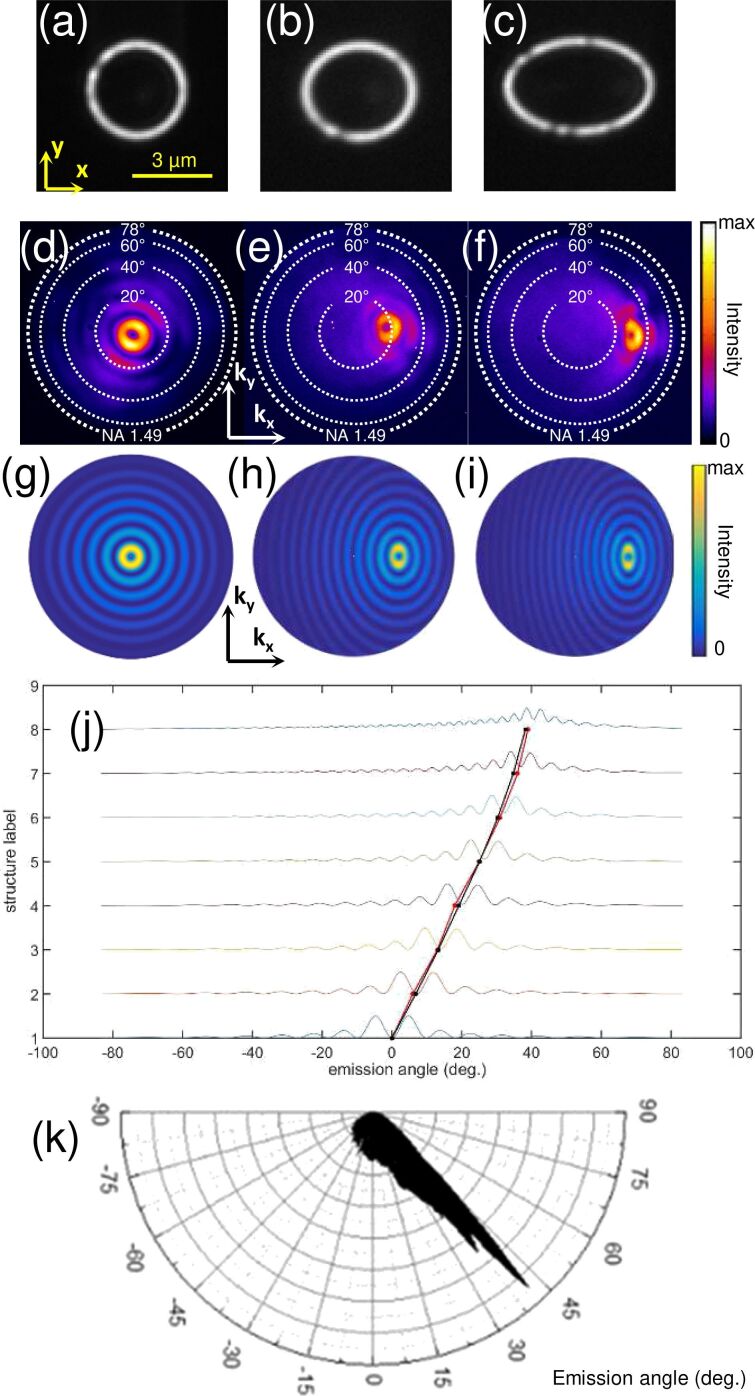
Control of the emission direction: effect of the eccentricity. (a–c) Transmission optical images under white light illumination, (d–f) experimental and (g–i) theoretical Fourier-space images of the light emitted upon electrical excitation of structures 1, 4 and 6, respectively (see Table 1). The STM tip is located in the center of structure 1, and at the focus *F*(*−c*,0) of structures 4 and 6. (j) Intensity profiles taken from theoretical Fourier-space images along **k_x_**, as well as the measured experimental emission angle (in red) and the calculated theoretical emission angle (in black) are shown. The calculated emission angle is determined from Equation 2. The intensity profiles are normalized and vertically offset for clarity. (k) Polar plot of the angular emission pattern retrieved from an experimental Fourier-space image of the light emitted upon electrical excitation of structure 8 (see Table 1) at the focus *F*(*−c*,0). Further experimental and theoretical Fourier-space images are provided in the Supporting Information File 1.

Figure 4 provides the experimental demonstration that light beams may be emitted in chosen directions from electrically driven elliptical slit antennas. The emission angle is determined by the eccentricity of the ellipse. Three different slits are used, i.e., structures 1, 4 and 6 in Table 1 (see also Figure 4a–c). They have eccentricities of 0 (circular slit), 0.51 and 0.77 (elliptical slits), respectively. The Fourier-space images recorded upon excitation with the tunnel electrons from the STM tip at one of the focii are shown in Figure 4d–f. Good agreement is found between the experimental data and the simulated images shown in Figure 4g–i. These simulated images are obtained using a model based on an ensemble of in-plane oscillating electric dipoles located along the slit and oriented orthogonally to the ellipse in the plane of the sample [45–46]. The same model was used to calculate the emission pattern from circular slits in [40]. Figure 4j shows the intensity profiles from the simulated Fourier-space images and the average emission angles retrieved from the experimental data for the eight elliptical slits the eccentricities of which are given in Table 1. The chosen eccentricity values are those corresponding to the intersection of a cylinder with a plane at angles of 0°, 10°…70°. On the same graph the theoretical value of the emission angle as obtained from Equation 2 is plotted, which reads


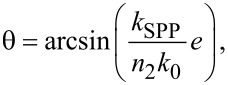


where (*k*_SPP_/*k*_0_) = 1.037 at λ_0_ = 700 nm and *n*_2_ = 1.518. In addition, Figure 4k shows a polar plot of the angular emission pattern retrieved from an experimental Fourier-space image of the light emitted upon electrical excitation of structure 8 (see Table 1) at the focus *F*(*−c*,0).

**Table 1:** Parameters of the elliptical slit antennas. The major axis 2*a* and the eccentricity *e* are given (the minor axis is always *2b* = 3 μm).

structure	1	2	3	4	5	6	7	8

2*a* (μm)	3	3.05	3.20	3.46	3.92	4.67	6	8.77
*e* (μm)	0	0.18	0.35	0.51	0.64	0.77	0.87	0.94
arcsin *e*	0°	10°	20°	30°	40°	50°	60°	70°

**Figure 5:**
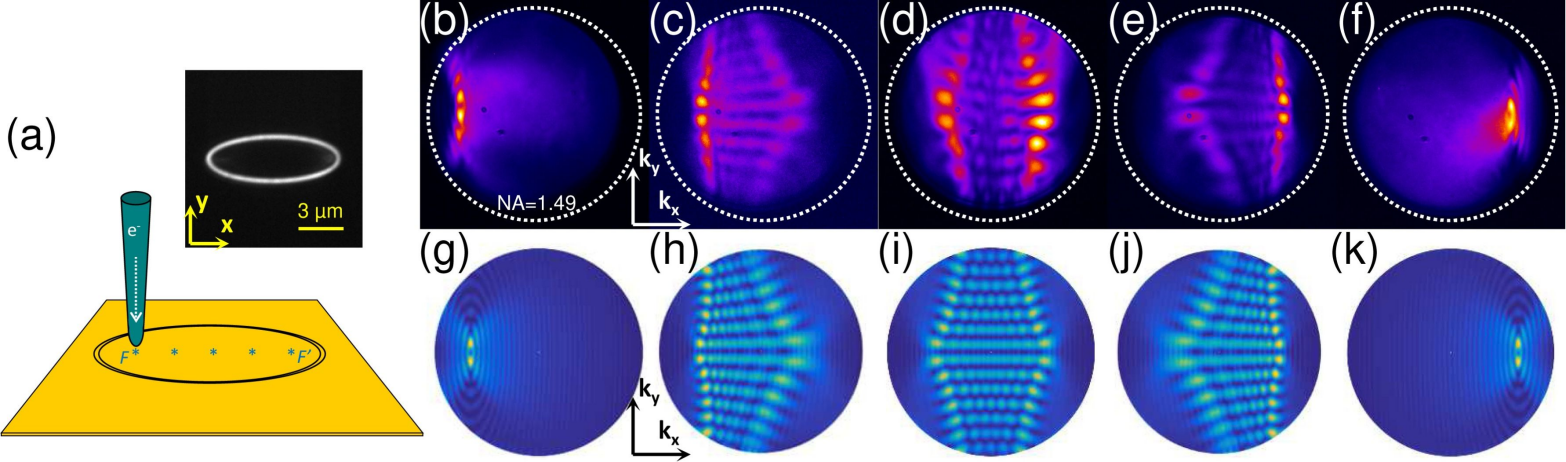
Angular emission pattern: effect of the excitation site. (a) Schematic of the experiment (inset: a transmission optical image under white light illumination). (b–f) Experimental and (g–k) theoretical Fourier-space images of the light emitted upon electrical excitation of structure 8 (see Table Table 1) at (from b to f) *F*′(*c*,0), (*c*/2,0), (0,0), (*−c*/2,0) and *F*(*−c*,0), respectively.

Figure 5 shows the effect of the SPP source location on the far-field emission pattern. The emission of light from an elliptical slit (structure 8 in Table 1) is obtained using the STM nanosource at different positions along the major axis of the ellipse, namely: on the focii *F*(*−c*,0) and *F*′(*c*,0), in the center *O*(0,0), and at intermediate positions, (*−c*/2,0) and (*c*/2,0). The corresponding experimental and theoretical Fourier-space images are shown in Figure 5. Intricated interference patterns, covering broad polar and azimuthal angular ranges, are produced when the SPP source is not located at one of the ellipse focii. Only when the excitation is located at a focus does the elliptical slit antenna yield angularly narrow light beams. As expected, the emission patterns upon excitation at *F* and *F*′ are perfect mirror symmetries of each other with respect to the (**yz**)-plane.

**Figure 6:**
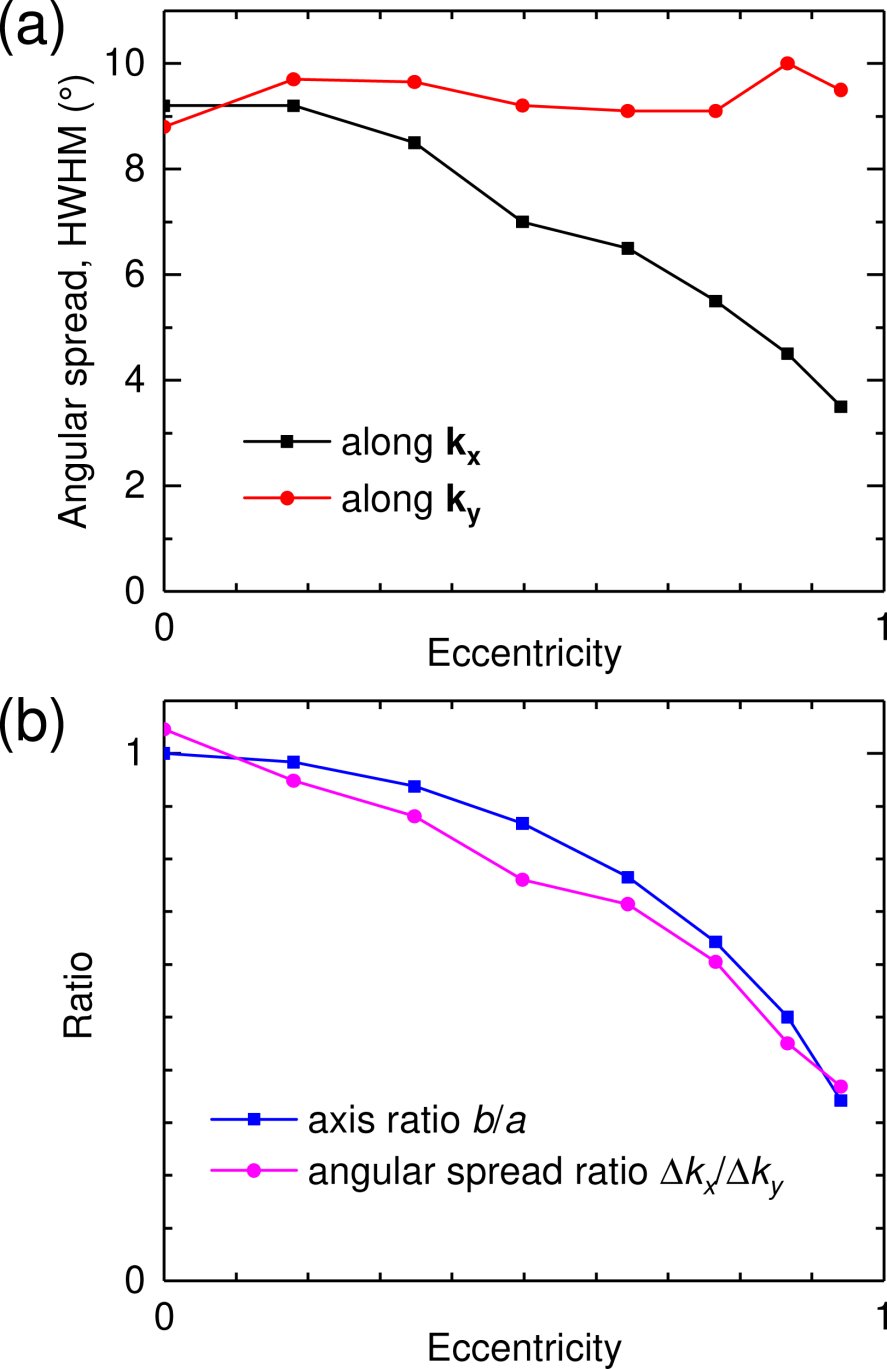
Angular spread: effect of the eccentricity. (a) Half width at half maximum (HWHM) of the angular distribution of the light emitted in glass. This data is obtained from experimental Fourier-space images along the *k_x_* and *k_y_* axes for structures 1–8. Here the HWHM is plotted as a function of the eccentricity of the ellipse. (b) Ratio of the minor and major semi-axes (*b*/*a*) and of the angular spread along *k_x_* and *k_y_* as a function of the ellipse eccentricity.

From the experimental Fourier-space images we retrieve the angular spread of the emitted light beams in the **k_x_** and **k_y_** reciprocal-space directions. Here we define the angular spread Δ*k* as the half width at half maximum (HWHM) of the light spot in the Fourier space. The results obtained for structures 1–8 (see Table 1) are plotted in Figure 6a as a function of the ellipse eccentricity. We see that the lateral size of the antenna determines the angular spread. The angular spread along **k_x_** decreases as the eccentricity increases since the major axis *2a* is increased while keeping the minor axis *2b* = 3 μm constant. For instance, angular spreads of 9° and of 4.5° along **k_x_** are measured for structure 1 (*2a* = 3μm) and 7 (*2a* = 6μm), respectively. No significant change occurs along **k_y_** where the angular spread remains within a range of 9–10°.

Figure 6b compares the angular spread ratio (Δ*k_x_*)/(Δ*k_y_*) to the minor-to-major axis ratio *b*/*a* of the ellipse. Close agreement is found between the two ratios. Due to the properties of Fourier transforms, the aspect ratio of the slit antenna in real space (*a*/*b*) and its emission pattern in Fourier space (Δ*k_x_*)/(Δ*k_y_*) are inversely proportional to each other. When elliptical slit antennas are fed at their focus, the angular spread of the emitted light beam is determined by the ellipse semiradii and the emission wavelength. It is worthy of note that despite higher SPP propagation losses is some directions, increasing *a* does not result in a broadening of the half width at half maximum along **k_x_**.

**Figure 7:**
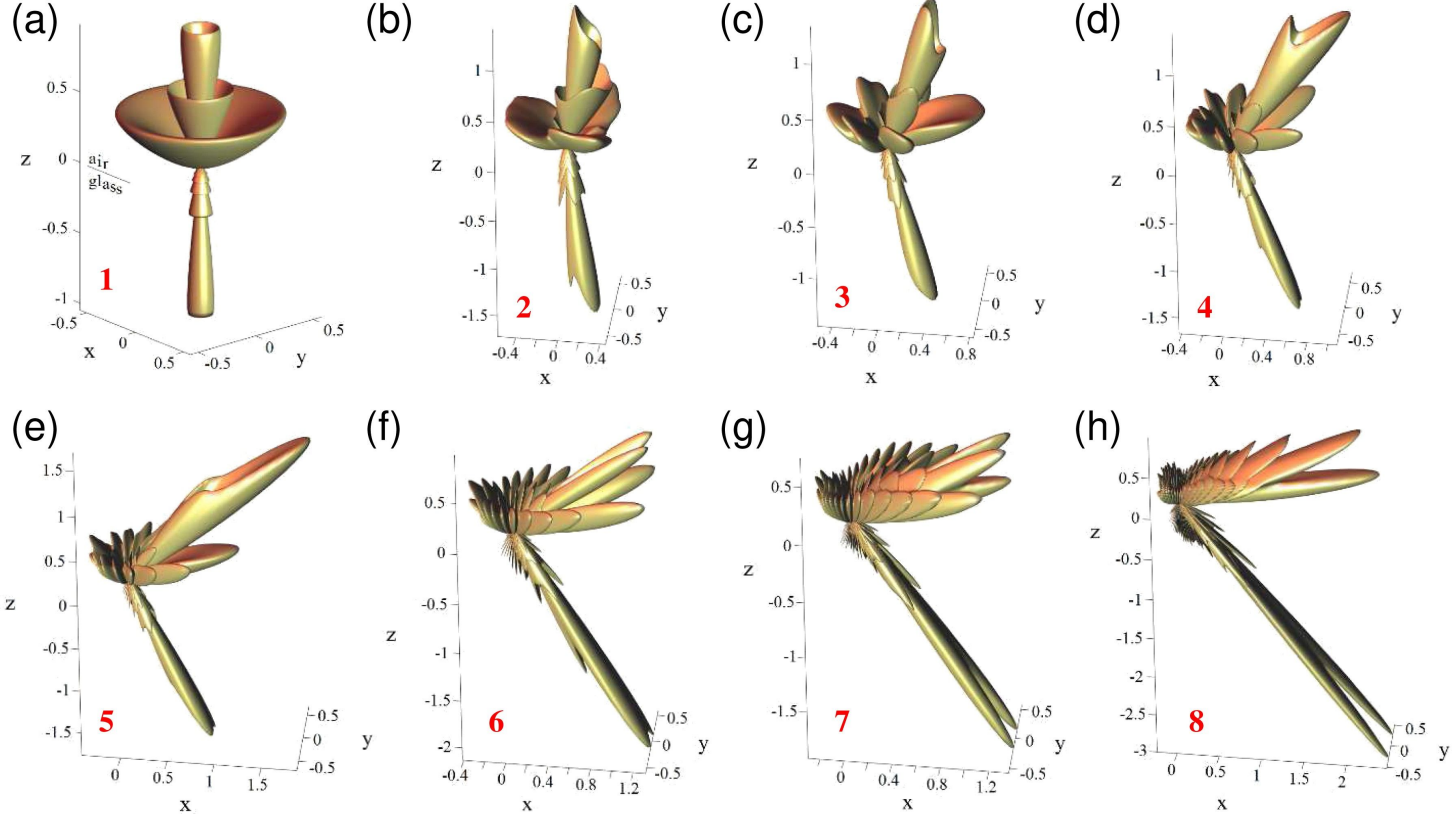
Lobe shapes. (a–h) Theoretical emission patterns from the elliptical slit antennas upon excitation by a **z**-oriented oscillating electric dipole located at the focus *F*(*−c*,0) of the ellipse. These patterns are calculated at a vacuum wavelength of λ_0_ = 700 nm for structures 1–8 (see Table 1).

**Figure 8:**
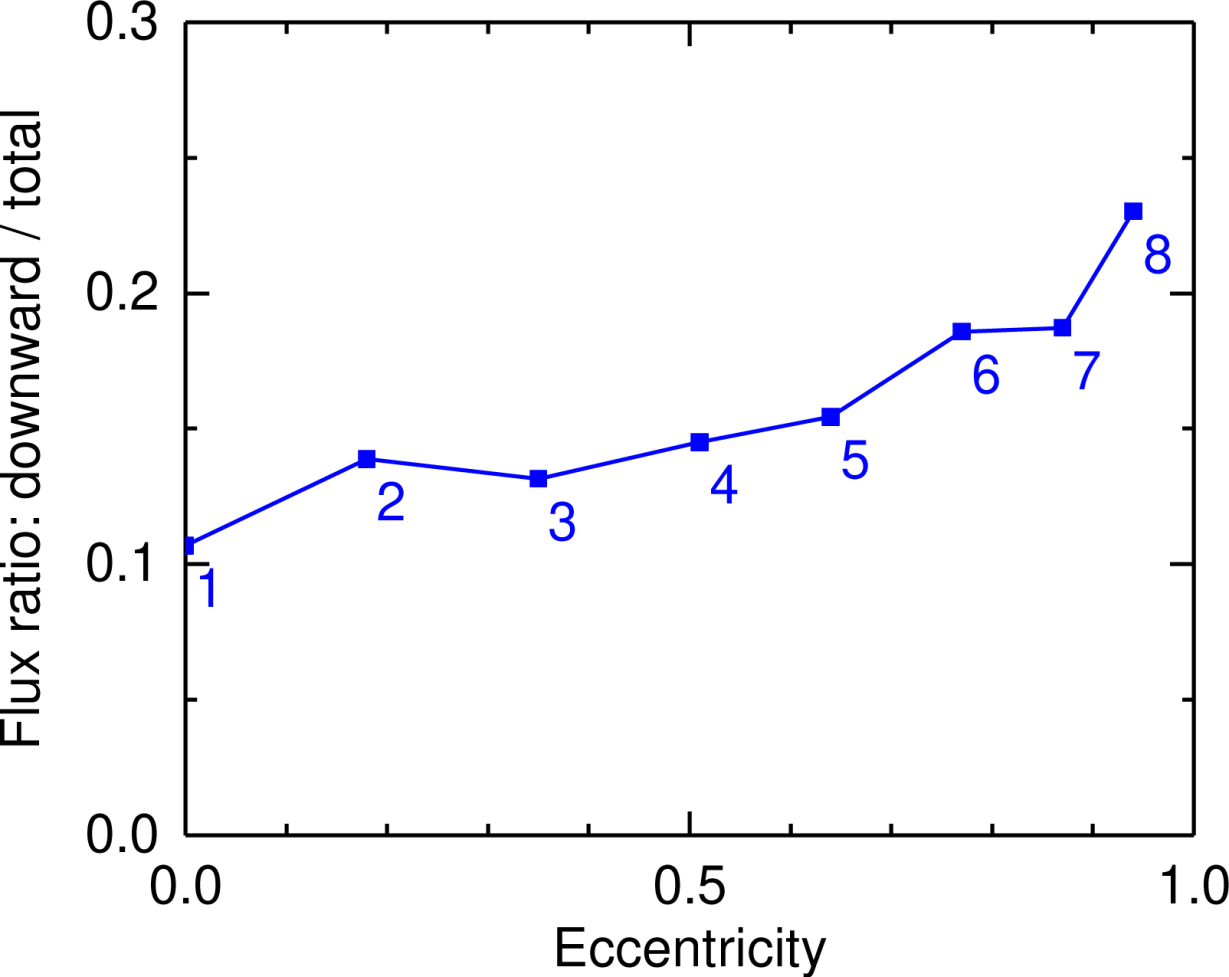
Where does the light go? Theoretical calculation of the Poynting vector: ratio of the light flux emitted downward (in the glass) to the total emitted flux (in air and glass) as calculated for structures 1–8 (see Table 1) from the diagrams shown in Figure 7. The data is plotted as a function of the ellipse eccentricity. Structure numbers are indicated next to the data points.

To further describe the emission from an elliptical slit antenna, we now examine the “shape” of the emission lobes as calculated at the vacuum wavelength of λ_0_ = 700 nm using the method described in [47–48]. In Figure 7a–h, the flux of the Poynting vector per unit solid angle is represented in real space for structures 1–8 (see Table 1). Electrical excitation at *F*(*−c*,0) is modeled as a monochromatic (λ_0_ = 700 nm) **z**-oriented oscillating dipole. Both the emission in the air (upward direction) and in the glass (downward direction) is shown. The air–glass interface is at *z* = 0 and the ellipse is centered at (*x*,*y*) = (0,0). In general, Figure 7 confirms that light beams are emitted both downward in the substrate and upward in the superstrate. The light is emitted at larger angles in the medium of lower refractive index (i.e., in air). Moreover, we see that a stronger beaming effect occurs in glass as compared to air. The light beam in glass has a lower angular spread and less intense side lobes. Even though higher directivity is obtained in glass with comparatively more light in the beaming direction than for the case in air, more intensity on average is emitted upward than downward. Figure 8 shows that the flux of the Poynting vector integrated over the lower half space represents only 10–25% of the total radiation (i.e., integrated over 4π sr).

### Field distribution in the slit

**Figure 9:**
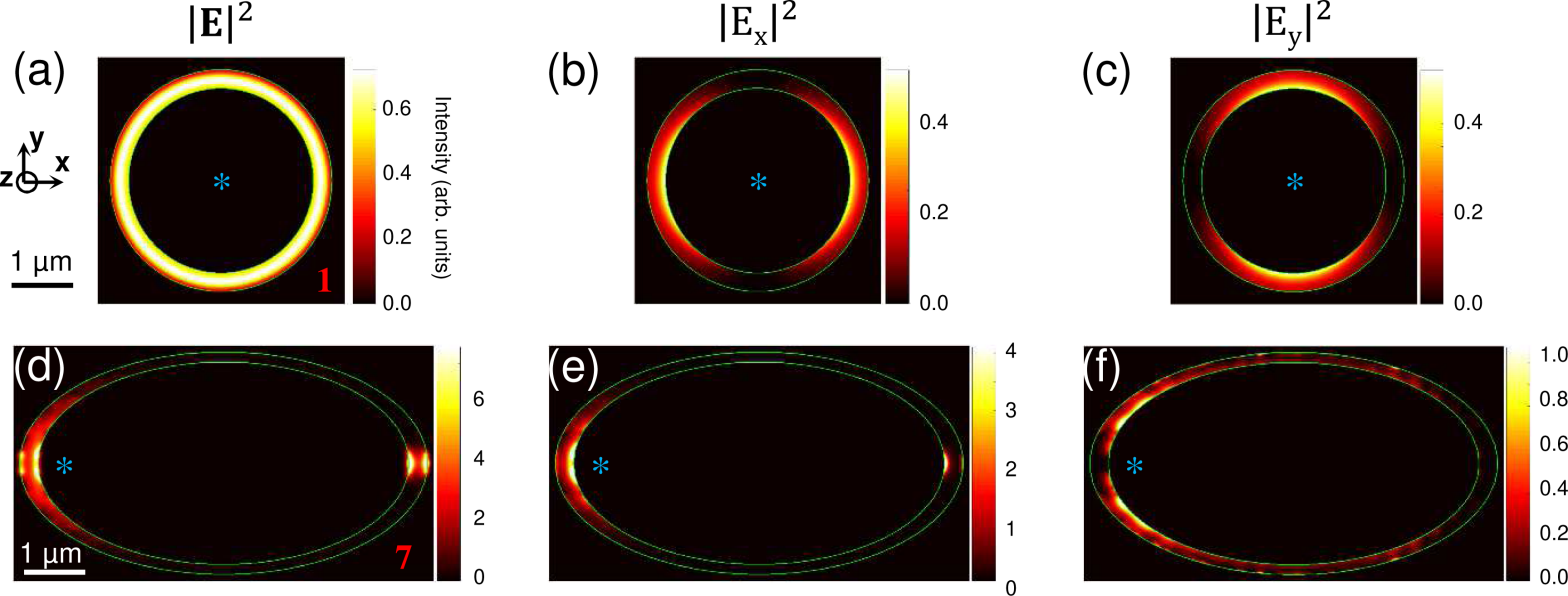
Theoretical **E**-field maps in the slit. (a–f) Spatial distribution of the square modulus of the total electric field |**E**|^2^ = |*E_x_*|^2^ + |*E_y_*|^2^ + |*E_z_*|^2^ and of its components |*E_x_*|^2^ and |*E_y_*|^2^ along the **x**- and **y**-axes, as calculated inside the slit of structures 1 (circular) and 7 (elliptical, see Table 1). The excitation is modeled as a monochromatic (λ_0_ = 700 nm) **z**-oriented oscillating electric dipole located at the center *F*(0,0) of the circular slit and at the “left focus” *F*(*−c*,0) of the elliptical slit (here, *c* = 2.60 μm). Both structures are centered at *O*(0,0). The dipole position is indicated with an asterisk.

Finally, we calculate the theoretical spatial distribution of the square modulus of the total electric field and its **x**- and **y**-components in the slit, i.e., in the (**xy**) plane, using the method described in [47–48]. Figure 9 shows the results for structures 1 (circular slit) and 7 (elliptical slit with *a*/*b* = 2, see Table 1). When SPPs are isotropically excited in the center of a circular slit antenna, the intensity of the scattered field inside the slit is spatially homogeneous (see Figure 9a) since the source-to-slit distance is the same for all points along the slit. The fact that the SPP propagation is orthogonal to the slit for all positions on the slit has two consequences: The SPP-to-light scattering efficiency is the same all along the slit, and the polarization of the scattered light is purely radial (see Figure 9b,c). This is in contrast to the elliptical slit antenna where the distance between the SPP source (located at one focus) and the slit depends on the direction. Therefore, the SPP amplitude decay due to propagation losses is direction-dependent as well, and the SPP amplitude at the slit depends on the location. Another effect that makes the SPP flux at the slit inhomogeneous (see Figure 9d) is the angle at which the SPP wavefront meets the slit. For the case of an ellipse and excitation at a focus, this angle will vary as a function of position on the slit. As a result, except along the major axis, the SPP wave does not meet the slit orthogonally. Consequently, the SPP-to-light scattering efficiency is likely to vary as a function of position along the slit.

In addition, the fact that the SPP propagation is not orthogonal to the slit has an effect on the polarization of the scattered light. SPP-to-light scattering at the slit essentially relies on the excitation of surface plasmons that oscillate in the plane of the sample in the direction perpendicular to the slit [45]. If this direction coincides with the radial direction for all positions along the slit (i.e., from the center of the structure to the slit), then the polarization of the scattered light is purely radial. This is true in the case of a circular slit; it is not true for an ellipse (see Figure 9e,f), where the radius and the direction of propagation only coincide along the major axis. Radial polarization yields a zero of intensity at the center of the light spot in Fourier space due to a polarization singularity along the propagation axis of the resulting light beam [49]. This zero of intensity is indeed observed in the experimental and theoretical data shown in Figure 4d and Figure 4g for the circular antenna. The Fourier-space images measured and calculated for the elliptical structures also exhibit a doughnut-shaped spot with a marked intensity dip, which, however, does not fall completely to zero. This confirms that the polarization is not purely radial and a combination of linear and radial polarization must occur with an increasing linear contribution as the ellipse eccentricity increases. Nevertheless, the radial component must dominate over the linear component even at an eccentricity as high as 0.94, otherwise no intensity dip would be seen. For symmetry reasons, the linear contribution to the polarization of the light beam must be oriented along the major axis of the ellipse (the off-center location of the SPP nanosource along the **x**-axis breaks the mirror symmetry of the system with respect to the **yz**-plane).

## Conclusion

We have introduced the working principle of an electrically driven elliptical slit antenna, which is a highly directive, low-energy, electrical microsource of light beams emitting in controlled directions. The emission direction is tailored by design by controlling the eccentricity of the ellipse. The model of a cylinder intersecting a plane may be used to describe the dependence of the beam direction and geometry on the structure eccentricity and the refractive index of the surrounding medium in a simple way. We have shown that the angular spread of the emitted beam depends on the length of the ellipse axes and not on its eccentricity. In addition, light beaming is robust to amplitude inhomogeneities of the scattered field from the slit (which becomes more inhomogeneous as the eccentricity increases) but is highly sensitive to its phase distribution (which changes when moving the SPP source from the ellipse focus). This is expected since light beaming from a slit is essentially a far-field interference effect. From calculations of the field in the slit, we infer that the polarization of the emitted beam is predominantly radial with a minor linear contribution (along the major axis) that increases with eccentricity. Future improvements of these optical antennas include the integration of the electrical SPP nanosource in the design of the microstructure (e.g., as an integrated metal-oxide–metal tunnel junction) and the engineering of the refractive indices of substrate and superstrate for greater control of the emission pattern, of the upward/downward power distribution and of the propagation losses. The principles of electrically driven elliptical slit antennas may be extended to similar chiral slit structures, e.g., elliptical spirals, in order to control the optical orbital angular moment of the emitted light beams [50–51].

## Experimental

**Figure 10:**
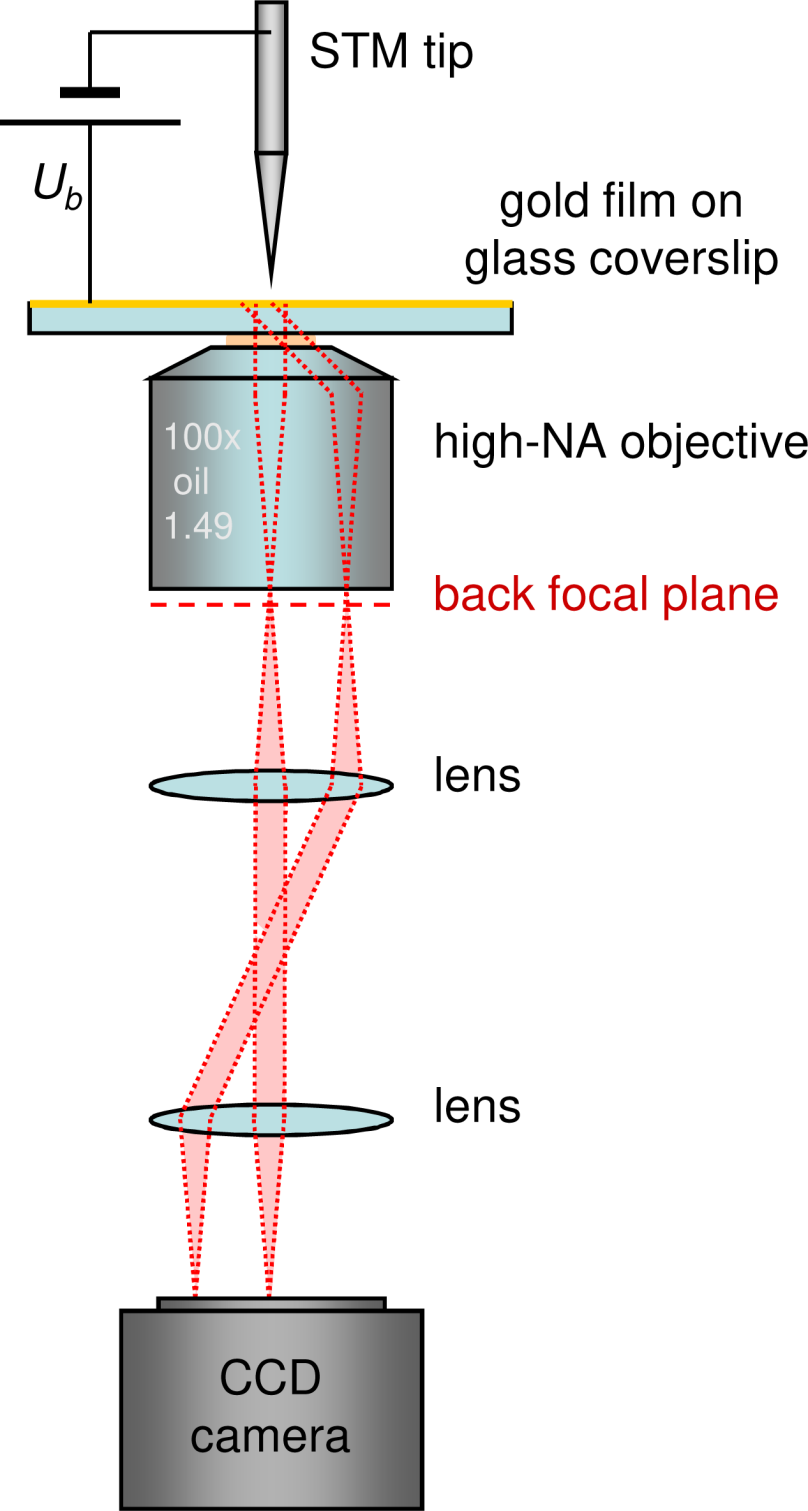
Schematic of the experimental setup. An STM head is mounted on top of an inverted optical microscope. The light emitted in the substrate upon electrical excitation of the sample with tunnel electrons from the tip is collected by the objective. The principle of Fourier-space imaging is illustrated with red dotted lines: parallel light rays emitted from the sample converge at the same point in the back focal plane of the objective. Thus, the angular distribution of the emitted light is retrieved from the image of the back focal plane on a CCD camera.

Figure 10 shows a schematic representation of the experimental setup. It consists of a commercial STM head (JPK Instruments, NanoWizard 3) mounted on top of an inverted optical microscope (Nikon Instruments, Eclipse Ti-U) equipped with a nanopositioning stage and an oil-immersion, high numerical aperture (NA = 1.49), 100× objective lens (Nikon CFI Apochromat TIRF objective). The STM is operated in air under ambient conditions [52]. STM tips are electrochemically etched tungsten wires.

A set of achromatic doublet lenses (Thorlabs, AC254-200-B) arranged in a 4*f* geometry is used to project an image of the back focal plane of the objective on a cooled CCD camera (Andor, IKON-M), to record the Fourier-space images (angular distribution of the collected light). The Fourier-space images shown in this paper are recorded under the following conditions: acquisition time 300 s, sample bias 2.8 V, setpoint current 1 nA.

The sample consists of a 200 nm thick gold film thermally evaporated on a standard, 170 μm thick, microscope glass coverslip coated with a transparent, 100 nm thick, conducting indium tin oxide (ITO) layer (purchased from SOLEMS, Palaiseau, France). ITO is used to electrically connect the inner gold area delineated by the elliptical slit to the rest of the gold film as is required for applying the tip–sample bias voltage for the STM measurements. The elliptical slits are milled in the gold film using a focused ion beam (FIB) at the NanoFab facility (Institut Néel, Grenoble, France). A scanning electron microscopy image of an elliptical slit is shown in Supporting Information File 1.

## Supporting Information

Additional experimental data, a scanning electron microscopy image of an elliptical slit and a description of the method to retrieve the angular spread from Fourier-space images are all provided.

File 1 Additional experimental data.
