# Supplementary material for: Directional light beams by design from electrically driven elliptical slit antennas
Source: Beilstein J Nanotechnol. 2018 Sep 3;9:2361–71. doi: 10.3762/bjnano.9.221 (PMC6142739; doi:10.3762/bjnano.9.221)
Supplement: File 1 — Additional experimental data. [file Beilstein_J_Nanotechnol-09-2361-s001.pdf]

Supporting Information

for

Directional light beams by design from electrically driven elliptical slit antennas

Shuiyan Cao[1], Eric Le Moal*[1], Quanbo Jiang[2], Aurélien Drezet[2], Serge Huant[2], Jean-Paul Hugonin[3], Gérald Dujardin[1] and Elizabeth Boer-Duchemin[1]

Address: [1]Institut des Sciences Moléculaires d'Orsay (ISMO), CNRS, Univ Paris Sud, Université Paris-Saclay, F-91405 Orsay (France); [2]Université Grenoble Alpes, Institut NEEL, F-38000 Grenoble, France and CNRS, Institut NEEL, F-38042 Grenoble, France and [3]Laboratoire Charles Fabry, Institut d'Optique, 91127 Palaiseau, France

Email: Eric Le Moal - eric.le-moal@u-psud.fr

* Corresponding author

Additional experimental data

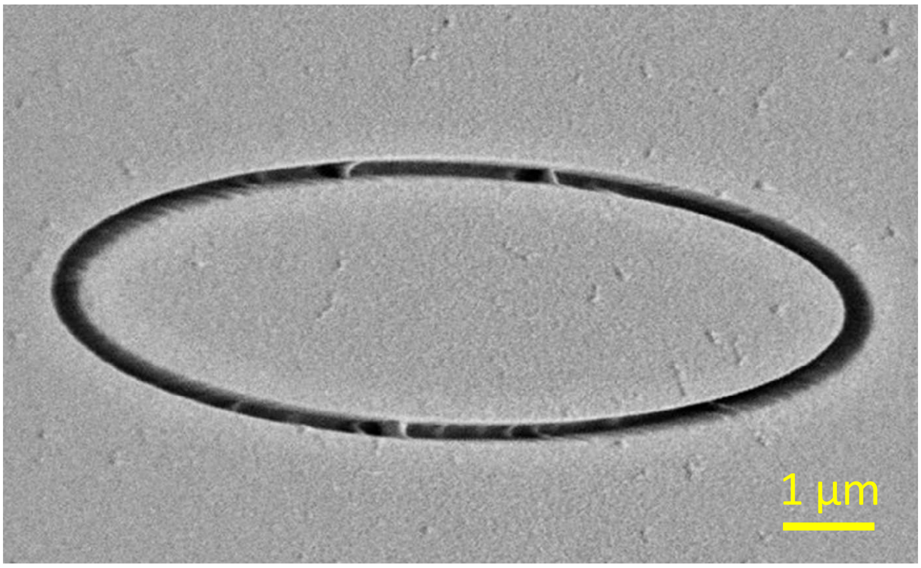

Figure S1: *The slit geometry.* Scanning electron microscopy image of an elliptical slit etched in a 20 0nm thick gold film (Structure 8 in the main text). The major and minor axes of the ellipse are $2a$ = 8.77 µm and $2b$ = 3 µm. The eccentricity of the ellipse is $e = 0.94$. The slit width is 300 nm and its depth is 200 nm. The small defects inside the slit are due to material redeposition during the focused-ion-beam sputtering process.

Figure S4 shows that light beams are emitted from electrically-driven elliptical slit antennas in directions that are determined by the ellipse eccentricity as long as the excitation is located at one of the ellipse focii. Figure S4(a1-a8) shows transmission optical images of elliptical slit antennas obtained under white light illumination. The slits are etched in a 200 nm thick gold film. These slits are referred to as Structures 1 to 8, respectively, and their geometrical parameters are given in Table 1 of the main text. Figure S4(b1-b8) shows schematics of the experiment for each structure. The tip of the scanning tunneling microscope is located at the "left focus" of each ellipse. Figure S4(c1-c8) shows the resulting Fourier-space optical microscopy images that are obtained when the electrical excitation is located at a focus as described in Figure S4(b1-b8). Figure S4(d1-d8) shows polar plots of intensity profiles obtained from the Fourier-space images along the dashed line shown in Figure S4(d1). The Fourier-space images are recorded under the following conditions: acquisition time 300 s, sample bias 2.8 V, setpoint current 1 nA.

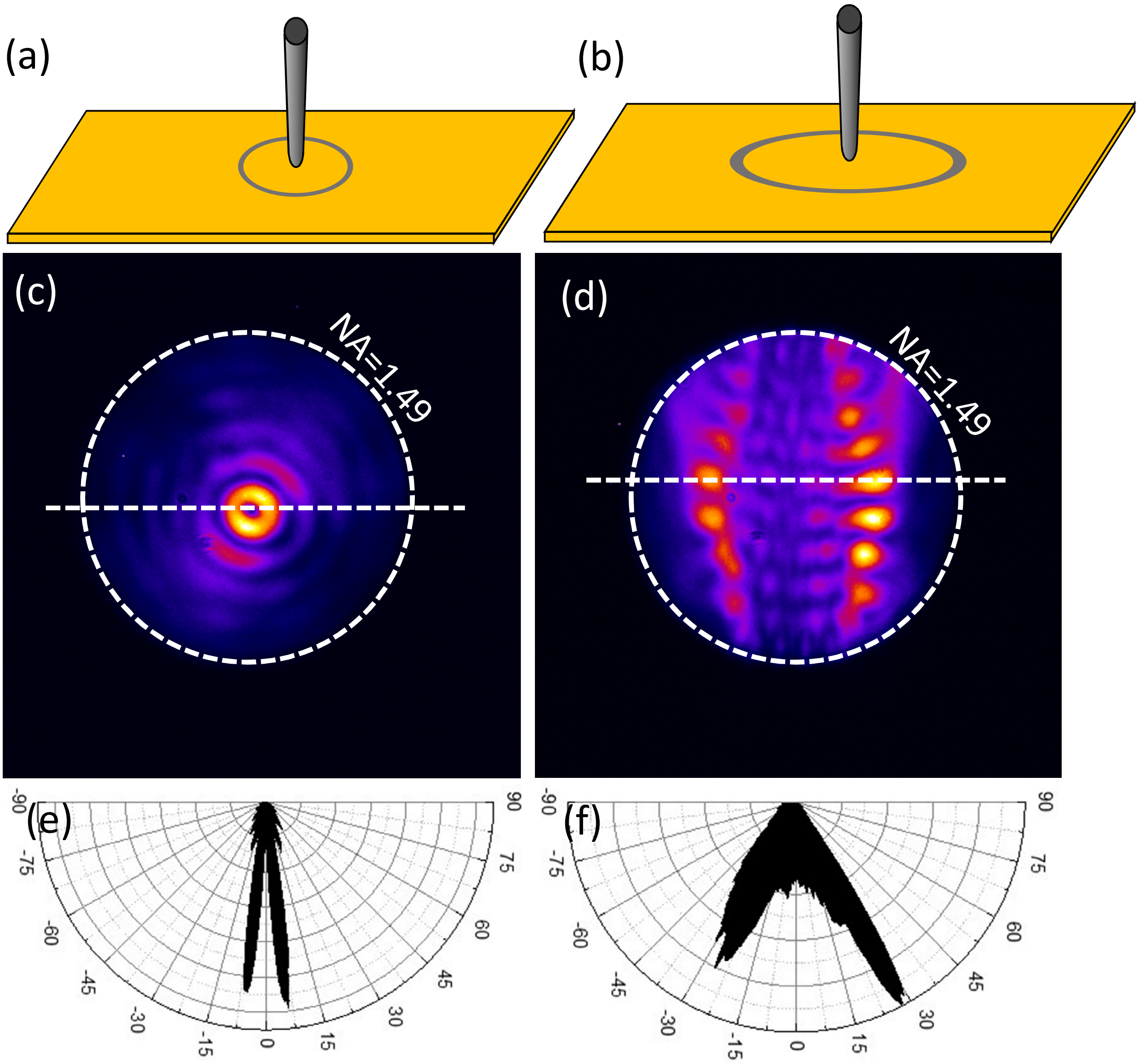

Figure S2: *Excitation in the center of the structure: circular and elliptical slit antennas.* (a,b) Schematics of the experiment: the tip of a scanning tunneling microscope (STM) is used to electrically excite surface plasmon polaritons (SPPs) at the center of (a) a circular slit antenna of diameter $2a$ = 3 µm and (b) an elliptical slit antenna with major and minor axes $2a$ = 8.77 µm and $2b$ = 3 µm etched in a 200 nm thick gold film. (c,d) Fourier-space optical microscopy images of the light scattered from the slit in the substrate, experimentally measured on (c) the circular slit antenna and (d) the elliptical slit antenna. (e,f) Polar plots of the intensity profiles obtained along the dashed lines on the Fourier-space images shown in (c) and (d), respectively. The Fourier-space images are recorded under the following conditions: acquisition time 300 s, sample bias 2.8 V, setpoint current 1 nA.

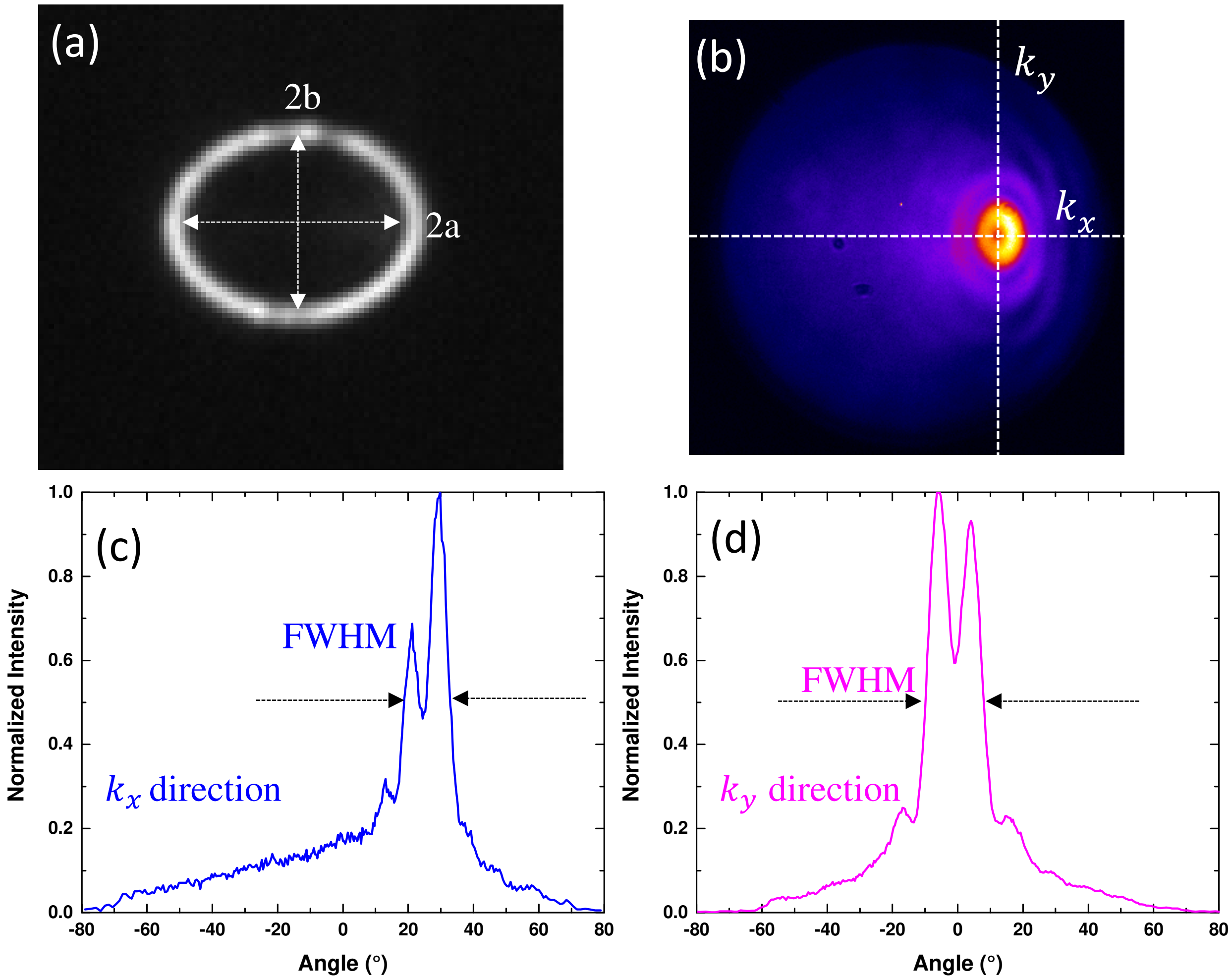

Figure S3: *Angular spread.* (a) Transmission optical image under white light illumination. The elliptical slit has major and minor axes $2a = 3.92$ µm and $2b = 3$ µm (Structure 5 in the main text). (b) Fourier-space image measured upon excitation of the elliptical slit antenna at its "left focus". (c,d) Intensity profiles taken from the Fourier-space image shown in (b) along the (c) $\mathbf{k_x}$ and (d) $\mathbf{k_y}$ directions, respectively. Arrows indicate the full width at half maximum (FWHM) of the intensity profiles. In the text, the angular spread is defined as the half width at half maximum. The Fourier-space image is recorded under the following conditions: acquisition time 300 s, sample bias 2.8 V, setpoint current 1 nA.

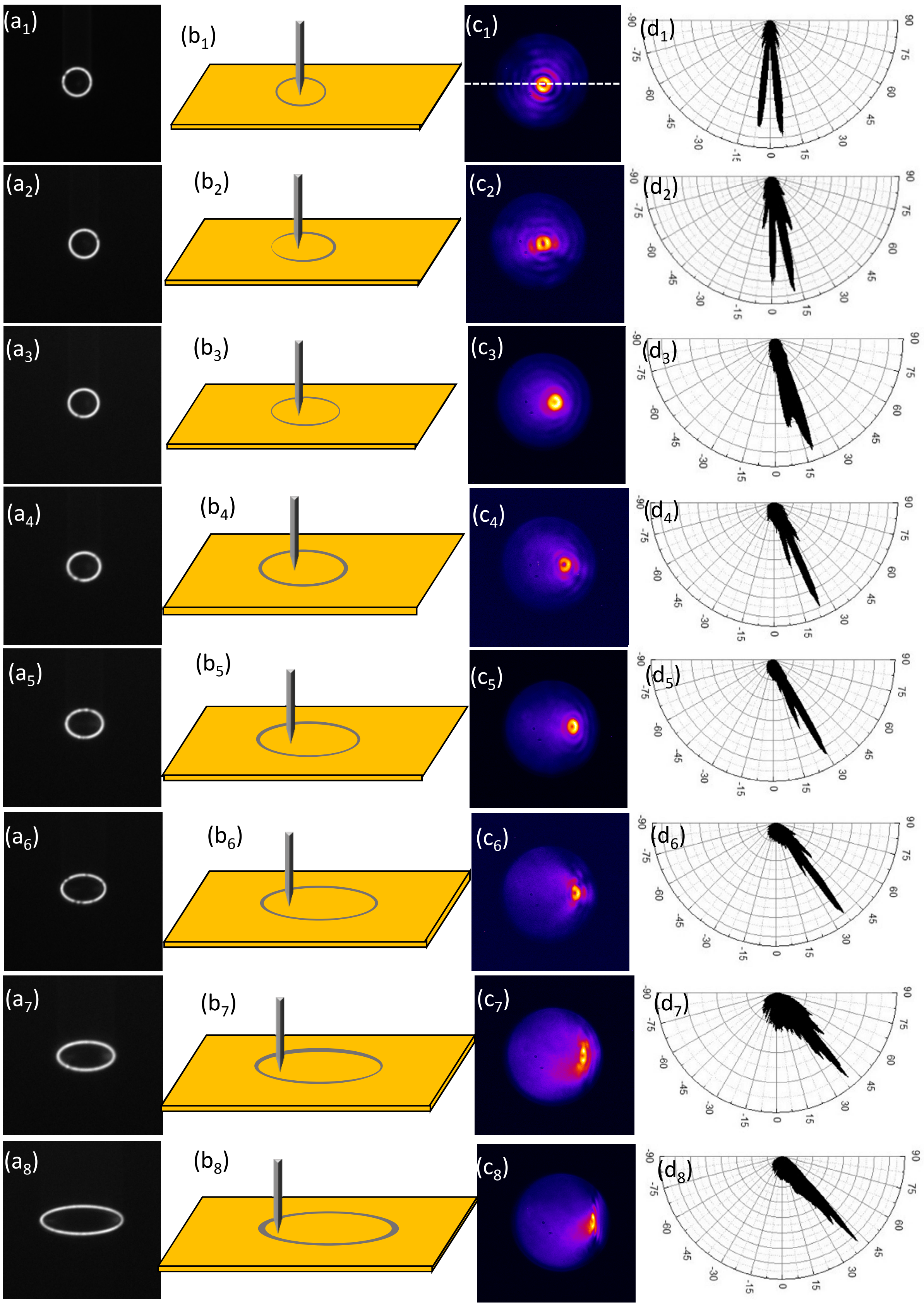

Figure S4: *Directivity control.* See page S2 for description of this figure.

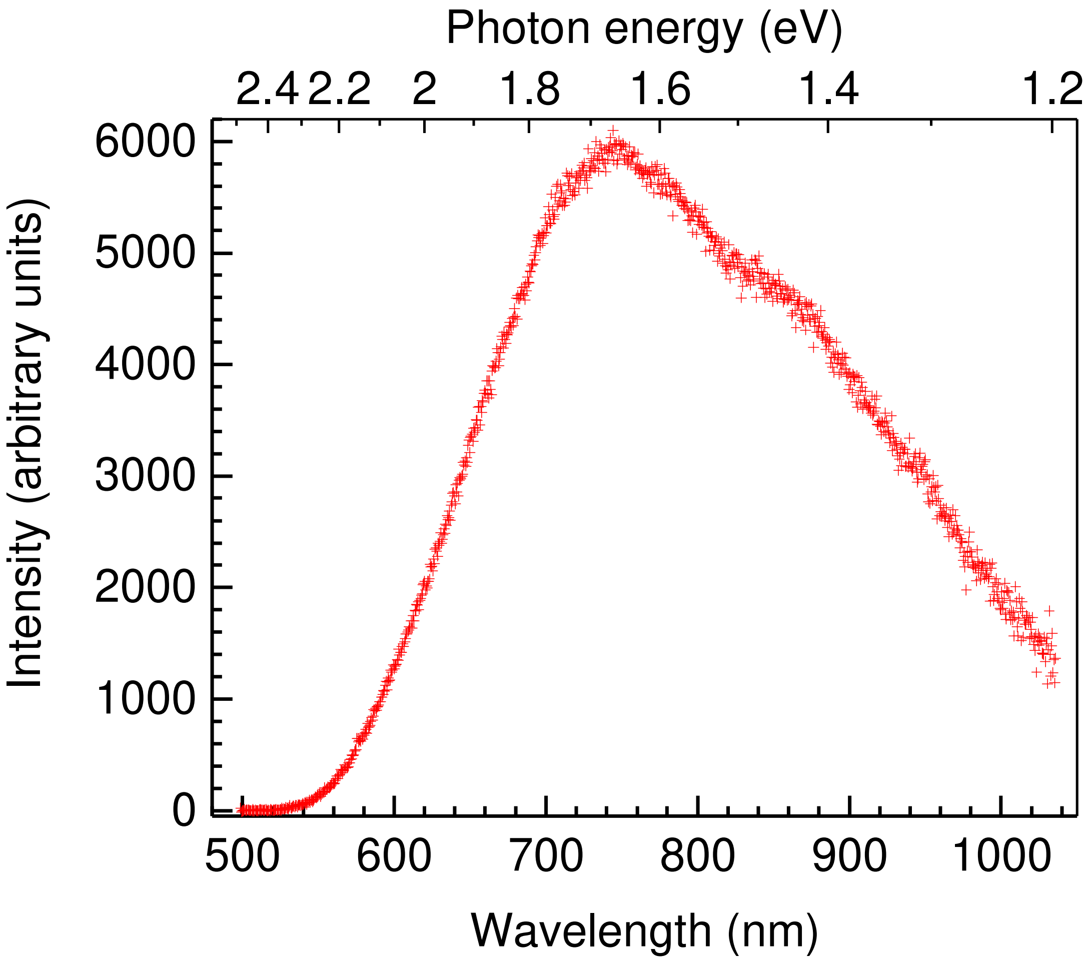

Figure S5: *Emission spectrum.* Experimentally measured spectral distribution of the light emitted upon electrical excitation in the center of structure 1 (see Table 1). The spectrum is corrected for the spectral response of the instrument, which includes the transmission of the microscope objective, the scattering efficiency of the diffraction grating and the quantum efficiency of the CCD camera used in the spectrometer. The STM bias is 2.8 V, the current setpoint is 3–4 nA and the acquisition time is 300 s.
